# Novel domain-specific POU3F4 mutations are associated with X-linked deafness: examples from different populations

**DOI:** 10.1186/s12881-015-0149-2

**Published:** 2015-02-25

**Authors:** Guney Bademci, Akeem O Lasisi, Kemal O Yariz, Paola Montenegro, Ibis Menendez, Rodrigo Vinueza, Rosario Paredes, Germania Moreta, Asli Subasioglu, Susan Blanton, Suat Fitoz, Armagan Incesulu, Levent Sennaroglu, Mustafa Tekin

**Affiliations:** John P. Hussmann Institute for Human Genomics and John T. Macdonald Foundation, Department of Human Genetics, Miller school of Medicine, University of Miami, 1501 NW 10th Avenue, BRB-610 (M-860), Miami, FL 33136 USA; Department of Otorhinolaryngology, College of Medicine, University of Ibadan, Ibadan, Nigeria; Departamento de Genetica, Hospital de Especialidades FFAA, Quito, Ecuador; Department of Medical Genetics, Izmir Katip Celebi University, Ataturk Training and Research Hospital, Izmir, Turkey; Department of Radiodiagnostics, Ankara University School of Medicine, Ankara, Turkey; Department of Otorhinolaryngology, Eskisehir Osmangazi University School of Medicine, Eskisehir, Turkey; Department of Otorhinolaryngology, Hacettepe University School of Medicine, Ankara, Turkey

**Keywords:** Molecular Diagnosis, Mutation Detection, *POU3F4*, Sequencing, X-linked deafness

## Abstract

**Background:**

Mutations in the *POU3F4* gene cause X-linked deafness type 3 (DFN3), which is characterized by inner ear anomalies.

**Methods:**

Three Turkish, one Ecuadorian, and one Nigerian families were included based on either inner ear anomalies detected in probands or X-linked family histories. Exome sequencing and/or Sanger sequencing were performed in order to identify the causative DNA variants in these families.

**Results:**

Four novel, c.707A>C (p.(Glu236Ala)), c.772delG (p.(Glu258ArgfsX30)), c.902C>T (p.(Pro301Leu)), c.987T>C (p.(Ile308Thr)), and one previously reported mutation c.346delG (p.(Ala116ProfsX26)) in *POU3F4*, were identified. All mutations identified are predicted to affect the POU-specific or POU homeo domains of the protein and co-segregated with deafness in all families.

**Conclusions:**

Expanding the spectrum of *POU3F4* mutations in different populations along with their associated phenotypes provides better understanding of their clinical importance and will be helpful in clinical evaluation and counseling of the affected individuals.

**Electronic supplementary material:**

The online version of this article (doi:10.1186/s12881-015-0149-2) contains supplementary material, which is available to authorized users.

## Background

Hearing loss is the most common sensory deficit with a prevalence of approximately 1 in 1000 newborns [1]. Genetic factors account for at least 50% of cases with congenital or prelingual-onset hearing loss in developed countries. Among the genetic forms, previous studies have indicated that deafness is transmitted with an inheritance pattern consistent with autosomal recessive in 75-77%, autosomal dominant in 15-20%, and X-linked in 2-3% of cases [2,3]. To date, five loci (DFNX1-4, and 6) and four genes, *PRPS1* (MIM 311850) for DFNX1 [4], *POU3F4* (MIM 300039) for DFNX2 [5], *SMPX* (MIM 300226) for DFNX4 [6,7], and *COL4A6* (MIM 303631) for DFNX6 have been identified for X-linked hearing loss [8].

DFNX2 (also referred to as Nance deafness) was originally described by Nance et al. in 1971 as an X-linked condition characterized in males by profound mixed deafness, vestibular abnormalities and congenital fixation at the stapes with perilymphatic gusher [9]. Computerized tomography studies in patients with DFNX2 showed abnormal dilatation of the internal acoustic canal and abnormal communication between the internal acoustic canal and inner ear compartments. Subsequently molecular analysis revealed mutations in *POU3F4* (POU domain, class III, transcription factor 4- NM_000307.4) [10].

*POU3F4* belongs to a superfamily of POU domain transcription factors comprised of a POU-specific domain and a POU-homeodomain, both of which influence DNA binding and specificity. Previous studies showed that the POU-specific part of the protein binds to DNA and regulates the downstream of the target genes [11]. The *POU3F4* gene is expressed in the developing neural tube during embryogenesis [11,12], and in a variety of other organs including the inner ear [13-17]. Fifty two mutations in *POU3F4* have been reported as the cause of DFNX2 since the initial report (Additional file 1: Table S1) [18]. These variants include intragenic mutations and partial or complete deletions of the gene, as well as chromosomal deletions, inversions, and duplications [19]. Here we report four novel and a previously reported mutations in the *POU3F4* gene identified in a multiethnic cohort of five families.

## Methods

### Subjects

This study was approved by the local Intitutional Review Board at the University of Miami (USA), the Ethics Commitee of Ankara University Medical School (Turkey), Bioethics Committee of FFAA (HE-1) in Quito (Ecuador), and the Joint Ethics Commitee of University of Ibadan/University College Hospital Ibadan (Nigeria). A signed informed consent form was obtained from each participant or in the case of a minor, from parents. In addition we obtained consent for publication of individual patient data from all of the study participants.

Diagnosis of sensorineural hearing loss was established via standard audiometry in a sound-proof room according to current clinical standards. A physical examination was performed on at least one hearing-impaired individual from each family including a detailed clinical evaluation and otoscopy. Affected individuals did not have delays in gross motor development. Neither did they have balance problems, vertigo, dizziness, or spontaneous and positional nystagmus. Tandem walking was normal and Romberg test was negative. Three Turkish families (295, 572, 667) were included based on inner ear anomalies detected in probands. One Ecuadorian (1225) and one Nigerian (1535) families were included based on X-linked family histories and physical examination that was negative for additional syndromic finding. Temporal bone CT scans were not available in probands from Ecuador and Nigeria.

### DNA Sequencing

Genomic DNA from total 5 affected and 8 unaffected family members was extracted from peripheral blood using standard methods. In probands from families 295, 572, 667, and 1535, the single exon of *POU3F4* was PCR-amplified and Sanger-sequenced. 20 ng of genomic DNA were used for PCR amplification. PCR products were purified by Qiagen MinElute PCR Purification Kit and directly sequenced with a Beckman Coulter CEQ2000XL or ABI 3730XL automated sequencers. In the proband of family 1225, exome sequencing was performed via Illumina HiSeq 2000 by using a previously published protocol [20]. Segregation of the identified variants was evaluated in the available family members of all families. DNA sequencing data are stored in a secure internal database, which are available upon request to researchers wishing to use them for research purposes only.

### Bioinformatics Analysis

For *in silico* analysis of the missense mutations, PolyPhen-2 (http://genetics.bwh.harvard.edu/pph2/index.shtml), SIFT (http://sift.jcvi.org/), and Mutation Taster (http://www.mutationtaster.org/) were used for the prediction of the effect of amino acid substitutions on protein function. In addition 3D structures were modeled on the Swiss-Model website (http://swissmodel.expasy.org) which generates modeling for the target protein based on a sequence alignment between the target protein and a suitable template structure. These models were visualized and delineated with RasMol (v. 2.7.5) software (http://rasmol.org/).

## Results and discussion

Five different *POU3F4* mutations were detected in the families (Table 1 and Additional file 2: Figure S1, S2, S3, S4 and S5). Detected variants co-segregated with hearing loss in all families. All four novel mutations were predicted to affect the POU domains of the protein. The novel NM_000307.4:c.772delG (p.(Glu258ArgfsX30)) mutation truncates the protein within POU-specific domain. Similarly, NM_000307.4:c.346delG (p.(Ala116ProfsX26)), which has been previously reported in a Korean family with severe deafness [21], leads to a truncation that eliminates both POU domains.

**Table 1:** **Clinical and genetic information of the probands in the affected families**

	**Family 295**	**Family 572**	**Family 667**	**Family 1225**	**Family 1535**
**Country origin**	Turkey	Turkey	Turkey	Ecuador	Nigeria
***POU3F4***	c.772delG	c.346delG	c.707A>C	c.902C>T	c.987T>C
**Mutation**	p.(Glu258ArgfsX30)	p.(Ala116Profsx26)	p.(Glu236Ala)	p.(Pro301Leu)	p.(Ile308Thr)
**Age of onset**	Congenital	<5 years	<5 years	<5 years	<5 years
**Pattern of inheritance**	Multiplex-X linked	Simplex	Simplex	Multiplex-X linked	Multiplex-X linked
**Hearing loss type**	Sensorineural	Sensorineural	Sensorineural	Mixed	Mixed
**Hearing loss severity**	Moderate to severe	Severe	Profound	Severe	Severe
**Laterality**	Bilateral	Bilateral	Bilateral	Bilateral	Bilateral
**Inner Ear Anomaly**	Basal turns of cochlea are incompletely separated from IAC which was dilated in the lateral end; modioli are absent	Dilated IAC, absence of modioli, interscalar septum is present	Bulbous enlargement of IAC; modioli are absent	N/A	N/A
**Intervention**	Hearing aids	Cochlear implant	Hearing aids	Hearing aids	Hearing aids

**Figure 1:**
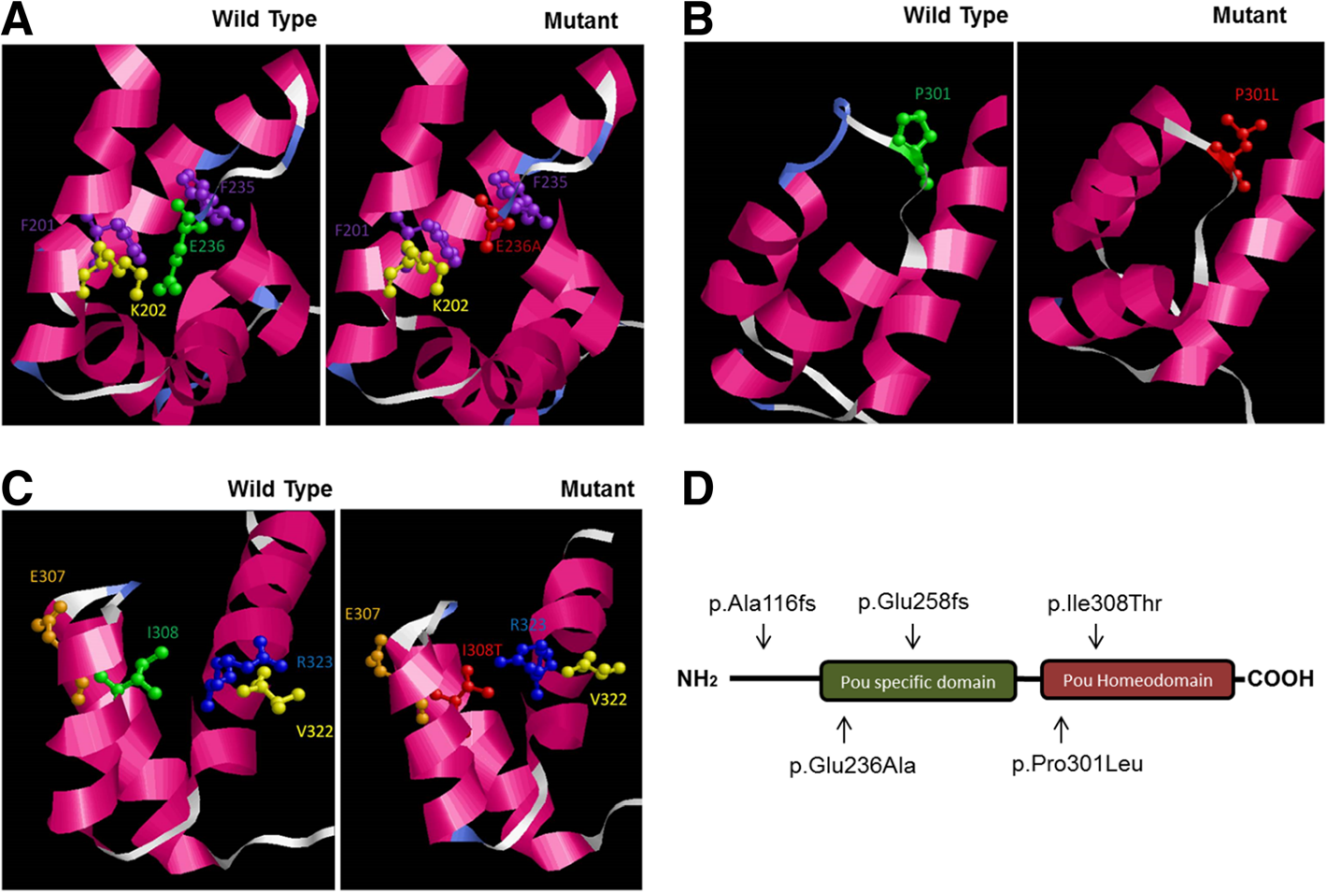
***In silico*** **analysis and molecular modeling of the wild-type and mutant POU3F4 proteins.** The p.Glu236Ala (p.E236A) mutation possibly interferes with the stability between the secondary structures of the protein **(A)**. The p.Pro301Leu (p.P301L) mutation affects the structure of the turn and the turn becomes shorter **(B)**. The hydroxide group introduced by the p.Ile308Thr (p.I308T) mutation causes a conformational change in the Arginine residue at 323 **(C)**. Schematic illustration of POU3F4 protein labeled with the five mutations identified in this study **(D)**.

*In silico* analysis for the three missense mutations, NM_000307.4:c.707A>C (p.(Glu236Ala)), c.902C>T (p.(Pro301Leu)), c.987T>C (p.(Ile308Thr)), predicted the effect of amino acid substitutions on protein function classified as pathogenic (PolyPhen-2: probably damaging; SIFT: damaging; Mutation Taster: disease causing). Structural analysis of the p.Glu236Ala mutation showed that the wild-type glutamate residue has a possible stabilizing relationship with the lysine residue (Lys202) residing within the alpha helix across the glutamate. The p.Glu236Ala mutation introduces a non-polar amino acid (alanine) instead of an acidic residue and possibly interferes with the stability between the secondary structures of the protein (Figure 1A). Proline has a distinctive cyclic structure, which provides a conformational rigidity. For that reason, it can commonly be found at turns in protein structures, as seen here in the POU homeodomain. In the p.Pro301Leu mutation, the structure of the turn changes becoming shorter (Figure 1B). The wild-type isoleucine at position 308 is part of an alpha-helix in the POU homeodomain. The p.Ile308Thr mutation introduces a threonine residue with a hydroxide group and, according to the model, the hydroxide group causes a conformational change in the arginine residue at 323 (Figure 1C). All three missense mutations are located in the two POU domains of the protein (Figure 1D). None of the identified variants was present in the Exome Variant Server (http://evs.gs.washington.edu/EVS/), 1000 Genomes, dbSNP, Leiden Open Variation Database (LOVD), or Deafness Variation Database (DVD). These variants were also absent in our next-generation sequencing database that contains data of more than 2000 samples from different ethnicities including Turkish (>200), Latin American (>200), and Nigeria (>100).

The phenotype of Nance deafness occurs because of the loss of function mutations in the *POU3F4* gene, which encodes a transcription factor consisting of two highly conserved domains [22]. The 76-78 amino acids that contain the POU-specific domain cooperate with the POU-homedomain to enhance the binding affinity and specificity of DNA binding [16]. Hemizygous males and heterozygous females carrying *POU3F4* mutations present with different clinical features. The hearing loss affects males with rapid progression to severe or profound deafness within the prelingual period. Temporal bone CT scans of males with *POU3F4* mutations show inner ear anomalies. Males have increased perilymphatic pressure, which is responsible for gusher during stapes surgery. The female carriers of *POU3F4* mutations usually show no or slight hearing loss [23]. In this study only males were affected while carrier females did not present with any clinical findings. Affected males’ middle and inner ear anomalies were within the expected spectrum.

*POU3F4* gene product is localized in the nucleus and has a critical role in inner ear morphogenesis. Recent studies showed that *POU3F4* mutations might cause a shift of Pou3f4 nuclear localization to the cytoplasm [24]. All mutations we identified affect a POU domain suggesting that loss of DNA binding capability may be a common pathological mechanism for function [21].

## Conclusions

In the present study we conclude that mutations in *POU3F4* cause X-linked hearing loss in different populations. It is especially important that the Ecuadorian and Nigerian families were studied solely based on their inheritance pattern and found to have mutations in *POU3F4*, suggesting that it is a common cause of X-linked hearing loss worldwide. We recommend that diagnostic molecular laboratories prioritize developing a *POU3F4* test that would be offered even when the results of CT scans were not available. Identification of a causative mutation for deafness provides families with a chance to make informed reproductive decisions.

## Additional files

Additional file 1: Table S1. Reported *POU3F4* mutations. Additional file 2: Figure S1. Data of the Family 295. Chromatograms showing wild type (1A), hemizygous NM_000307.4:c.772delG (p.(Glu258ArgfsX30)) mutation in the affected male (1B) and c.772delG (p.(Glu258ArgfsX30)) mutation in the female carrier (1C). The pedigree contains two affected male siblings (1D). **Figure S2.** Data of the family 667. Chromatograms showing wild type (2A), hemizygous NM_000307.4:c.707A>C (p.(Glu236Ala)) mutation in the affected male (2B) and c.707A>C (p.(Glu236Ala)) mutation in the female carrier (2C). One affected male was in the family (2D). **Figure S3.** Data of the family 572. Chromatograms showing wild type (3A), hemizygous NM_000307.4:c.346delG (p.(Ala116Profsx26)) mutation in the affected male (3B) and c.346delG (p.(Ala116Profsx26)) mutation in the female carrier (3C). One affected male was in the family (3D). **Figure S4.** Data of the family 1225. Chromatograms showing wild type (4A), hemizygous NM_000307.4:c.902C>T (p.(Pro301Leu)) mutation in the affected male (4B) and c.902C>T (p.(Pro301Leu)) mutation in the female carrier (4C). The pedigree contains five affected males and is consistent with X-linked recessive inheritance (4D). **Figure S5.** Data of the family 1535. Chromatogram showing hemizygous NM_000307.4:c.987T>C (p.(Ile308Thr)) mutation in the affected male (5A). There are three affected males and the pedigree is consistent with X-Linked recessive inheritance (5B).

## Abbreviations

CT: Computerized tomography
IAC: Internal Acoustic Canal
N/A: Not Available
