# Supplementary material for: Novel domain-specific POU3F4 mutations are associated with X-linked deafness: examples from different populations
Source: BMC Med Genet. 2015 Feb 25;16:9. doi: 10.1186/s12881-015-0149-2 (PMC4422282; doi:10.1186/s12881-015-0149-2)
Supplement: Additional file 1: Table S1. — Reported POU3F4 mutations. [file 12881_2015_149_MOESM1_ESM.doc]

| **Missense/nonsense** | | |
| --- | --- | --- |
| **Protein** | **Nucleotide** | **Reference** |
| p.W67X | c.200G>A | [Cremers (2000) Adv Otorhinolaryngol 56, 184](http://www.ncbi.nlm.nih.gov/sites/entrez?cmd=Retrieve&db=PubMed&list_uids=10868234&dopt=Abstract) |
| p.Q79X | c.235C>T | Parzefall (2013) Hum Mutat 34, 1102 |
| p.S98X | c.293C>A | [Marlin (2009) Clin Genet 76, 558](http://www.ncbi.nlm.nih.gov/sites/entrez?cmd=Retrieve&db=PubMed&list_uids=19930154&dopt=Abstract) |
| p.W114 | c.341G>A | Waryah (2011) J Hum Genet 56, 534 |
| p.Q136 | c.406C>T | [Waryah (2011) J Hum Genet 56, 534](http://www.ncbi.nlm.nih.gov/sites/entrez?cmd=Retrieve&db=PubMed&list_uids=21633365&dopt=Abstract) |
| p.R167X | c.499C>T | [Stankovic (2010) Ann Otol Rhinol Laryngol 119, 815](http://www.ncbi.nlm.nih.gov/sites/entrez?cmd=Retrieve&db=PubMed&list_uids=21250553&dopt=Abstract) |
| p.K202X | c.604A>T | [de Kok (1995) Science 267, 685](http://www.ncbi.nlm.nih.gov/sites/entrez?cmd=Retrieve&db=PubMed&list_uids=7839145&dopt=Abstract) |
| p.L208X | c.623T>A | [Lee (2009) Clin Genet 75, 572](http://www.ncbi.nlm.nih.gov/sites/entrez?cmd=Retrieve&db=PubMed&list_uids=19438930&dopt=Abstract) |
| p.T211M | c.632C>T | [Choi (2013) Hum Mutat 34, 309](http://www.ncbi.nlm.nih.gov/sites/entrez?cmd=Retrieve&db=PubMed&list_uids=23076972&dopt=Abstract) |
| p.G216E | c.647G>A | [Li (2010) J Genet Genomics 37, 787](http://www.ncbi.nlm.nih.gov/sites/entrez?cmd=Retrieve&db=PubMed&list_uids=21193157&dopt=Abstract) |
| p.S228L | c.683C>T | [Vore (2005) Arch Otolaryngol Head Neck Surg 131, 1057](http://www.ncbi.nlm.nih.gov/sites/entrez?cmd=Retrieve&db=PubMed&list_uids=16365218&dopt=Abstract) |
| p.Q229R | c.686A>G | [Choi (2013) Hum Mutat 34, 309](http://www.ncbi.nlm.nih.gov/sites/entrez?cmd=Retrieve&db=PubMed&list_uids=23076972&dopt=Abstract) |
| p.T230I | c.689C>T | [Friedman (1997) Ann Otol Rhinol Laryngol 106, 320](http://www.ncbi.nlm.nih.gov/sites/entrez?cmd=Retrieve&db=PubMed&list_uids=9109724&dopt=Abstract) |
| p.P303S | c.907C>T | [Cremers (2000) Adv Otorhinolaryngol 56, 184](http://www.ncbi.nlm.nih.gov/sites/entrez?cmd=Retrieve&db=PubMed&list_uids=10868234&dopt=Abstract) |
| p.I308N | c.923T>A | [Marlin (2009) Clin Genet 76, 558](http://www.ncbi.nlm.nih.gov/sites/entrez?cmd=Retrieve&db=PubMed&list_uids=19930154&dopt=Abstract) |
| p.S309P | c.925T>C | [Wang (2006) Laryngoscope 116, 944](http://www.ncbi.nlm.nih.gov/sites/entrez?cmd=Retrieve&db=PubMed&list_uids=16735904&dopt=Abstract) |
| p.A312V | c.935C>T | [Bitner-Glindzicz (1995) Hum Mol Genet 4, 1467](http://www.ncbi.nlm.nih.gov/sites/entrez?cmd=Retrieve&db=PubMed&list_uids=7581392&dopt=Abstract) |
| p.L317W | c.950T>G | [de Kok (1995) Science 267, 685](http://www.ncbi.nlm.nih.gov/sites/entrez?cmd=Retrieve&db=PubMed&list_uids=7839145&dopt=Abstract) |
| p.R323G | c.967C>G | [Cremers (2000) Adv Otorhinolaryngol 56, 184](http://www.ncbi.nlm.nih.gov/sites/entrez?cmd=Retrieve&db=PubMed&list_uids=10868234&dopt=Abstract) |
| p.W325R | c.973T>A | [Schild (2011) Otol Neurotol epub](http://www.ncbi.nlm.nih.gov/sites/entrez?cmd=Retrieve&db=PubMed&list_uids=21555964&dopt=Abstract) |
| p.N328T | c.983A>C | [Cremers (2000) Adv Otorhinolaryngol 56, 184](http://www.ncbi.nlm.nih.gov/sites/entrez?cmd=Retrieve&db=PubMed&list_uids=10868234&dopt=Abstract) |
| p.R329P | c.986G>C | [Lee (2009) Physiol Genomics 39, 195](http://www.ncbi.nlm.nih.gov/sites/entrez?cmd=Retrieve&db=PubMed&list_uids=19671658&dopt=Abstract) |
| p.R329G | c.985C>G | [Friedman (1997) Ann Otol Rhinol Laryngol 106, 320](http://www.ncbi.nlm.nih.gov/sites/entrez?cmd=Retrieve&db=PubMed&list_uids=9109724&dopt=Abstract) |
| p.R330S | c.990A>T | [de Kok (1997) Hum Mutat 10, 207](http://www.ncbi.nlm.nih.gov/sites/entrez?cmd=Retrieve&db=PubMed&list_uids=9298820&dopt=Abstract) |
| p.K334E | c.1000A>G | [de Kok (1995) Science 267, 685](http://www.ncbi.nlm.nih.gov/sites/entrez?cmd=Retrieve&db=PubMed&list_uids=7839145&dopt=Abstract) |
| p.X362R | c.1084T>C | [Choi (2013) Hum Mutat 34, 309](http://www.ncbi.nlm.nih.gov/sites/entrez?cmd=Retrieve&db=PubMed&list_uids=23076972&dopt=Abstract) |
| **Small deletions/insertions** | | |
| **Nucleotide** | | **Reference** |
| c.346del1 | | [Lee (2009) Physiol Genomics 39, 195](http://www.ncbi.nlm.nih.gov/sites/entrez?cmd=Retrieve&db=PubMed&list_uids=19671658&dopt=Abstract) |
| c.383del1 | | [Lee (2009) Clin Genet 75, 572](http://www.ncbi.nlm.nih.gov/sites/entrez?cmd=Retrieve&db=PubMed&list_uids=19438930&dopt=Abstract) |
| c.607_610del4 | | [de Kok (1995) Science 267, 685](http://www.ncbi.nlm.nih.gov/sites/entrez?cmd=Retrieve&db=PubMed&list_uids=7839145&dopt=Abstract) |
| c.601_606del6 | | [Hagiwara (1998) Laryngoscope 108, 1544](http://www.ncbi.nlm.nih.gov/sites/entrez?cmd=Retrieve&db=PubMed&list_uids=9778298&dopt=Abstract) |
| c.648del1 | | [de Kok (1995) Science 267, 685](http://www.ncbi.nlm.nih.gov/sites/entrez?cmd=Retrieve&db=PubMed&list_uids=7839145&dopt=Abstract) |
| c.853_854delAT | | [Parzefall (2013) Hum Mutat 34, 1102](http://www.ncbi.nlm.nih.gov/sites/entrez?cmd=Retrieve&db=PubMed&list_uids=23606368&dopt=Abstract) |
| c.862_865del4 | | [Bitner-Glindzicz (1995) Hum Mol Genet 4, 1467](http://www.ncbi.nlm.nih.gov/sites/entrez?cmd=Retrieve&db=PubMed&list_uids=7581392&dopt=Abstract) |
| c.896del1 | | [de Kok (1995) Science 267, 685](http://www.ncbi.nlm.nih.gov/sites/entrez?cmd=Retrieve&db=PubMed&list_uids=7839145&dopt=Abstract) |
| c.927_929del3 | | [Lee (2009) Physiol Genomics 39, 195](http://www.ncbi.nlm.nih.gov/sites/entrez?cmd=Retrieve&db=PubMed&list_uids=19671658&dopt=Abstract) |
| c.1060delA | | [Choi (2013) Hum Mutat 34, 309](http://www.ncbi.nlm.nih.gov/sites/entrez?cmd=Retrieve&db=PubMed&list_uids=23076972&dopt=Abstract) |
| c.950dupT | | Choi (2013) Hum Mutat 34, 309 |
| **Gross deletions/insertions** | | |
| **Description** | | **Reference** |
| 1-1.5Mb located 90kb upstream of ge (described at genomic DNA level) | | [Song (2010) Clin Genet 78, 524](http://www.ncbi.nlm.nih.gov/sites/entrez?cmd=Retrieve&db=PubMed&list_uids=20412083&dopt=Abstract) |
| 120 kb, upstream of gene (described at genomic DNA level) | | [de Kok (1996) Hum Mol Genet 5, 1229](http://www.ncbi.nlm.nih.gov/sites/entrez?cmd=Retrieve&db=PubMed&list_uids=8872461&dopt=Abstract) |
| 15-17Mb incl entire gene, RSK4 & se (described at genomic DNA level) | | [Song (2010) Clin Genet 78, 524](http://www.ncbi.nlm.nih.gov/sites/entrez?cmd=Retrieve&db=PubMed&list_uids=20412083&dopt=Abstract) |
| 200 kb, upstream of gene (described at genomic DNA level) | | [de Kok (1996) Hum Mol Genet 5, 1229](http://www.ncbi.nlm.nih.gov/sites/entrez?cmd=Retrieve&db=PubMed&list_uids=8872461&dopt=Abstract) |
| 220 kb, upstream of gene (described at genomic DNA level) | | [de Kok (1996) Hum Mol Genet 5, 1229](http://www.ncbi.nlm.nih.gov/sites/entrez?cmd=Retrieve&db=PubMed&list_uids=8872461&dopt=Abstract) |
| 30 kb, upstream of gene (described at genomic DNA level) | | [de Kok (1996) Hum Mol Genet 5, 1229](http://www.ncbi.nlm.nih.gov/sites/entrez?cmd=Retrieve&db=PubMed&list_uids=8872461&dopt=Abstract) |
| 3902 bp, upstream of gene (described at genomic DNA level) | | [Naranjo (2010) Hum Genet 128, 411](http://www.ncbi.nlm.nih.gov/sites/entrez?cmd=Retrieve&db=PubMed&list_uids=20668882&dopt=Abstract)  [Ahn (2009) Genesis 47: 137 [Additional report]](http://www.ncbi.nlm.nih.gov/sites/entrez?cmd=Retrieve&db=PubMed&list_uids=19217071&dopt=Abstract) |
| 5.6Mb incl entire gene (described at genomic DNA level) | | [Marlin (2009) Clin Genet 76, 558](http://www.ncbi.nlm.nih.gov/sites/entrez?cmd=Retrieve&db=PubMed&list_uids=19930154&dopt=Abstract) |
| 8 kb, upstream of gene (described at genomic DNA level) | | [de Kok (1996) Hum Mol Genet 5, 1229](http://www.ncbi.nlm.nih.gov/sites/entrez?cmd=Retrieve&db=PubMed&list_uids=8872461&dopt=Abstract) |
| entire gene (described at genomic DNA level) | | [de Kok (1996) Hum Mol Genet 5, 1229](http://www.ncbi.nlm.nih.gov/sites/entrez?cmd=Retrieve&db=PubMed&list_uids=8872461&dopt=Abstract) |
| ~650 kb incl. entire gene (described at genomic DNA level) | | [Vore (2005) Arch Otolaryngol Head Neck Surg 131, 1057](http://www.ncbi.nlm.nih.gov/sites/entrez?cmd=Retrieve&db=PubMed&list_uids=16365218&dopt=Abstract) |
| Duplication of 150 kb, 170 kb upstream of gene (described at genomic DNA level) | | [de Kok (1995) Hum Mol Genet 4, 2145](http://www.ncbi.nlm.nih.gov/sites/entrez?cmd=Retrieve&db=PubMed&list_uids=8589693&dopt=Abstract) |
| **Complex** | | |
| **Description** | | **Reference** |
| 100-300 kb 5' gene + 1 Mb proximal | | [Oh (2003) HNO 51, 629](http://www.ncbi.nlm.nih.gov/sites/entrez?cmd=Retrieve&db=PubMed&list_uids=12942177&dopt=Abstract) |
| Inversion (X)(q21.1q22.3) | | Anger (2013) Am J Audiol epub |
| Six deletions of 2.6 kb, 6.5 kb, 2.7 kb, 6.5 kb, 7 kb, 4.4kb | | [de Kok (1996) Hum Mol Genet 5, 1229](http://www.ncbi.nlm.nih.gov/sites/entrez?cmd=Retrieve&db=PubMed&list_uids=8872461&dopt=Abstract) |

**Table S1:** Reported *POU3F4* mutations.

**References:**

Cremers FP, Cremers CW, Ropers HH.The ins and outs of X-linked deafness type 3. Adv Otorhinolaryngol 2000, 56:184-95.

Parzefall T, Shivatzki S, Lenz DR, Rathkolb B, Ushakov K, Karfunkel D, Shapira Y, Wolf M, Mohr M, Wolf E, Sabrautzki S, de Angelis MH, Frydman M, Brownstein Z, Avraham KB.Cytoplasmic mislocalization of POU3F4 due to novel mutations leads to deafness in humans and mice. Hum Mutat. 2013, 34:1102-10.

Marlin S, Moizard MP, David A, Chaissang N, Raynaud M, Jonard L, Feldmann D, Loundon N, Denoyelle F, Toutain A. [Phenotype and genotype in females with POU3F4 mutations.](http://www.ncbi.nlm.nih.gov/pubmed/19930154) Clin Genet. 2009, 76:558-63.

Waryah AM, Ahmed ZM, Bhinder MA, Choo DI, Sisk RA, Shahzad M, Khan SN, Friedman TB, Riazuddin S, Riazuddin S.

Molecular and clinical studies of X-linked deafness among Pakistani families.J Hum Genet. 2011, 56:534-40.

Stankovic KM, Hennessey AM, Herrmann B, Mankarious LA.Cochlear implantation in children with congenital X-linked deafness due to novel mutations in POU3F4 gene.Ann Otol Rhinol Laryngol. 2010, 119:815-22.

[de Kok YJ](http://www.ncbi.nlm.nih.gov/sites/entrez?Db=pubmed&Cmd=Search&Term="de Kok YJ"%5BAuthor%5D&itool=EntrezSystem2.PEntrez.Pubmed.Pubmed_ResultsPanel.Pubmed_RVAbstractPlusDrugs1), [van der Maarel SM](http://www.ncbi.nlm.nih.gov/sites/entrez?Db=pubmed&Cmd=Search&Term="van der Maarel SM"%5BAuthor%5D&itool=EntrezSystem2.PEntrez.Pubmed.Pubmed_ResultsPanel.Pubmed_RVAbstractPlusDrugs1), [Bitner-Glindzicz M](http://www.ncbi.nlm.nih.gov/sites/entrez?Db=pubmed&Cmd=Search&Term="Bitner-Glindzicz M"%5BAuthor%5D&itool=EntrezSystem2.PEntrez.Pubmed.Pubmed_ResultsPanel.Pubmed_RVAbstractPlusDrugs1), [Huber I](http://www.ncbi.nlm.nih.gov/sites/entrez?Db=pubmed&Cmd=Search&Term="Huber I"%5BAuthor%5D&itool=EntrezSystem2.PEntrez.Pubmed.Pubmed_ResultsPanel.Pubmed_RVAbstractPlusDrugs1), [Monaco AP](http://www.ncbi.nlm.nih.gov/sites/entrez?Db=pubmed&Cmd=Search&Term="Monaco AP"%5BAuthor%5D&itool=EntrezSystem2.PEntrez.Pubmed.Pubmed_ResultsPanel.Pubmed_RVAbstractPlusDrugs1), [Malcolm S](http://www.ncbi.nlm.nih.gov/sites/entrez?Db=pubmed&Cmd=Search&Term="Malcolm S"%5BAuthor%5D&itool=EntrezSystem2.PEntrez.Pubmed.Pubmed_ResultsPanel.Pubmed_RVAbstractPlusDrugs1), [Pembrey ME](http://www.ncbi.nlm.nih.gov/sites/entrez?Db=pubmed&Cmd=Search&Term="Pembrey ME"%5BAuthor%5D&itool=EntrezSystem2.PEntrez.Pubmed.Pubmed_ResultsPanel.Pubmed_RVAbstractPlusDrugs1), [Ropers HH](http://www.ncbi.nlm.nih.gov/sites/entrez?Db=pubmed&Cmd=Search&Term="Ropers HH"%5BAuthor%5D&itool=EntrezSystem2.PEntrez.Pubmed.Pubmed_ResultsPanel.Pubmed_RVAbstractPlusDrugs1), [Cremers FP](http://www.ncbi.nlm.nih.gov/sites/entrez?Db=pubmed&Cmd=Search&Term="Cremers FP"%5BAuthor%5D&itool=EntrezSystem2.PEntrez.Pubmed.Pubmed_ResultsPanel.Pubmed_RVAbstractPlusDrugs1). Association between X-linked mixed deafness and mutations in the POU domain gene POU3F4. Science. 1995, 267:685-8.

Lee HK, Song MH, Kang M, Lee JT, Kong KA, Choi SJ, Lee KY, Venselaar H, Vriend G, Lee WS, Park HJ, Kwon TK, Bok J, Kim UK.Clinical and molecular characterizations of novel POU3F4 mutations reveal that DFN3 is due to null function of POU3F4 protein.Physiol Genomics. 2009, 39:195-201.

Choi BY, Kim DH, Chung T, Chang M, Kim EH, Kim AR, Seok J, Chang SO, Bok J, Kim D, Oh SH, Park WY.Destabilization and mislocalization of POU3F4 by C-terminal frameshift truncation and extension mutation.Hum Mutat. 2013, 34:309-16.

Li J, Cheng J, Lu Y, Lu Y, Chen A, Sun Y, Kang D, Zhang X, Dai P, Han D, Yuan H. Identification of a novel mutation in POU3F4 for prenatal diagnosis in a Chinese family with X-linked nonsyndromic hearing loss.J Genet Genomics. 2010, 37:787-93.

Vore AP, Chang EH, Hoppe JE, Butler MG, Forrester S, Schneider MC, Smith LL, Burke DW, Campbell CA, Smith RJ.Deletion of and novel missense mutation in POU3F4 in 2 families segregating X-linked nonsyndromic deafness.Arch Otolaryngol Head Neck Surg. 2005, 131:1057-63.

Friedman RA, Bykhovskaya Y, Tu G, Talbot JM, Wilson DF, Parnes LS, Fischel-Ghodsian N.Molecular analysis of the POU3F4 gene in patients with clinical and radiographic evidence of X-linked mixed deafness with perilymphatic gusher.Ann Otol Rhinol Laryngol. 1997, 106:320-5.

Wang QJ, Li QZ, Rao SQ, Zhao YL, Yuan H, Yang WY, Han DY, Shen Y.A novel mutation of POU3F4 causes congenital profound sensorineural hearing loss in a large Chinese family.Laryngoscope. 2006, 116:944-50.

Bitner-Glindzicz M1, Turnpenny P, Höglund P, Kääriäinen H, Sankila EM, van der Maarel SM, de Kok YJ, Ropers HH, Cremers FP, Pembrey M, et al.Further mutations in Brain 4 (POU3F4) clarify the phenotype in the X-linked deafness, DFN3.Hum Mol Genet. 1995, 4:1467-9.

Schild C, Prera E, Lüblinghoff N, Arndt S, Aschendorff A, Birkenhäger R.Novel mutation in the homeobox domain of transcription factor POU3F4 associated with profound sensorineural hearing loss. Otol Neurotol. 2011, 32:690-4.

de Kok YJ, Cremers CW, Ropers HH, Cremers FP.The molecular basis of X-linked deafness type 3 (DFN3) in two sporadic cases: identification of a somatic mosaicism for a POU3F4 missense mutation.Hum Mutat. 1997, 10:207-11.

Lee HK, Lee SH, Lee KY, Lim EJ, Choi SY, Park RK, Kim UK.Novel POU3F4 mutations and clinical features of DFN3 patients with cochlear implants.Clin Genet. 2009, 75:572-5.

Hagiwara H, Tamagawa Y, Kitamura K, Kodera K.A new mutation in the POU3F4 gene in a Japanese family with X-linked mixed deafness (DFN3).Laryngoscope. 1998, 108:1544-7.

Song MH, Lee HK, Choi JY, Kim S, Bok J, Kim UK.Clinical evaluation of DFN3 patients with deletions in the POU3F4 locus and detection of carrier female using MLPA. Clin Genet. 2010, 78:524-32.

Naranjo S, Voesenek K, de la Calle-Mustienes E, Robert-Moreno A, Kokotas H, Grigoriadou M, Economides J, Van Camp G, Hilgert N, Moreno F, Alsina B, Petersen MB, Kremer H, Gómez-Skarmeta JL. Multiple enhancers located in a 1-Mb region upstream of POU3F4 promote expression during inner ear development and may be required for hearing.

Hum Genet. 2010, 128:411-9.

Oh N, Kupka S, Mirghomizadeh F, Arold R, Zimmermann R, Blin N, Zenner HP, Pfister M.Clinical and molecular genetic analysis of monozygotic twins displaying stapes gusher syndrome (DFN3). HNO. 2003, 51:629-33.

Anger GJ, Crocker S, McKenzie K, Brown KK, Morton CC, Harrison K, MacKenzie JJ.X-linked deafness-2 (DFNX2) phenotype associated with a paracentric inversion upstream of POU3F4.Am J Audiol. 2014, 23:1-6.

de Kok YJ, Vossenaar ER, Cremers CW, Dahl N, Laporte J, Hu LJ, Lacombe D, Fischel-Ghodsian N, Friedman RA, Parnes LS, Thorpe P, Bitner-Glindzicz M, Pander HJ, Heilbronner H, Graveline J, den Dunnen JT, Brunner HG, Ropers HH, Cremers FP.Identification of a hot spot for microdeletions in patients with X-linked deafness type 3 (DFN3) 900 kb proximal to the DFN3 gene POU3F4.Hum Mol Genet. 1996, 5:1229-35.
