# Supplementary material for: Novel domain-specific POU3F4 mutations are associated with X-linked deafness: examples from different populations
Source: BMC Med Genet. 2015 Feb 25;16:9. doi: 10.1186/s12881-015-0149-2 (PMC4422282; doi:10.1186/s12881-015-0149-2)
Supplement: Additional file 2: Figure S1. — Data of the Family 295. Chromatograms showing wild type (1A), hemizygous NM_000307.4:c.772delG (p.(Glu258ArgfsX30)) mutation in the affected male (1B) and c.772delG (p.(Glu258ArgfsX30)) mutation in the female carrier (1C). The pedigree contains two affected male siblings (1D). Figure S2. Data of the family 667. Chromatograms showing wild type (2A), hemizygous NM_000307.4:c.707A>C (p.(Glu236Ala)) mutation in the affected male (2B) and c.707A>C (p.(Glu236Ala)) mutation in the female carrier (2C). One affected male was in the family (2D). Figure S3. Data of the family 572. Chromatograms showing wild type (3A), hemizygous NM_000307.4:c.346delG (p.(Ala116Profsx26)) mutation in the affected male (3B) and c.346delG (p.(Ala116Profsx26)) mutation in the female carrier (3C). One affected male was in the family (3D). Figure S4. Data of the family 1225. Chromatograms showing wild type (4A), hemizygous NM_000307.4:c.902C>T (p.(Pro301Leu)) mutation in the affected male (4B) and c.902C>T (p.(Pro301Leu)) mutation in the female carrier (4C). The pedigree contains five affected males and is consistent with X-linked recessive inheritance (4D). Figure S5. Data of the family 1535. Chromatogram showing hemizygous NM_000307.4:c.987T>C (p.(Ile308Thr)) mutation in the affected male (5A). There are three affected males and the pedigree is consistent with X-Linked recessive inheritance (5B). [file 12881_2015_149_MOESM2_ESM.pdf]

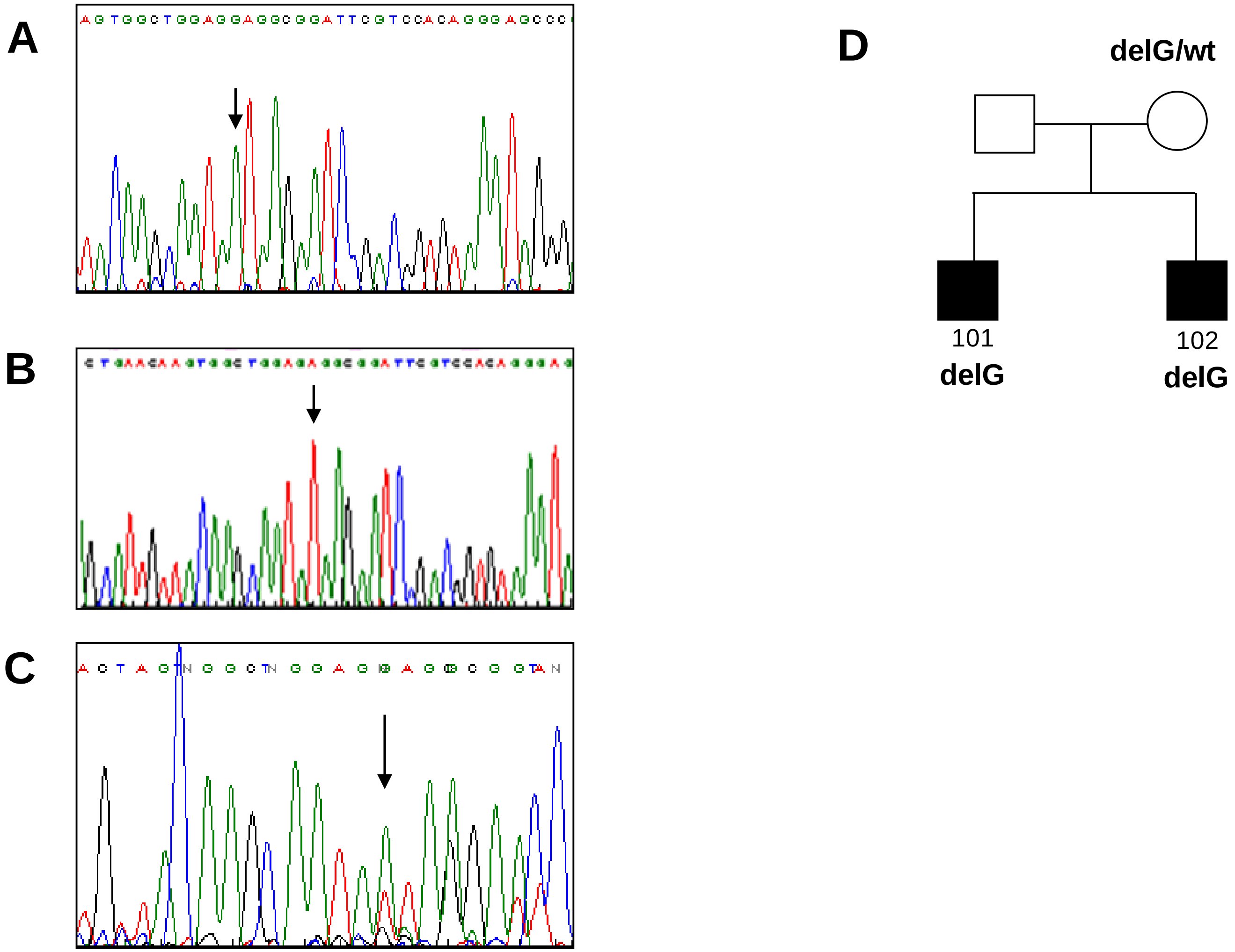

<u>Figure S1:</u>Data of the Family 295. Chromatograms showing wild type (1A), hemizygous NM_000307.4:c.772delG (p.(Glu258ArgfsX30)) mutation in the affected male (1B) and c.772delG (p.(Glu258ArgfsX30)) mutation in the female carrier (1C). The pedigree contains two affected male siblings (1D).

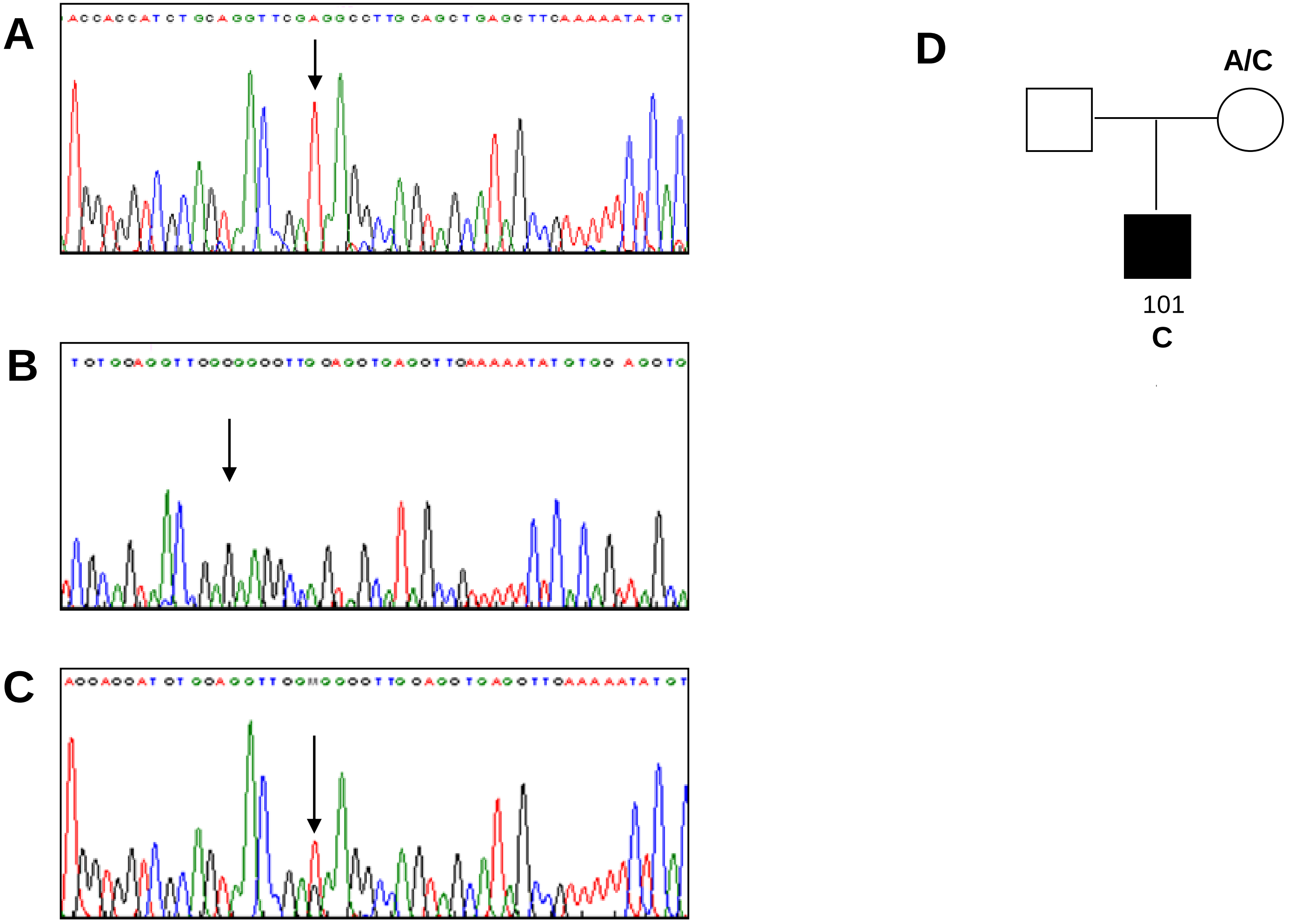

Figure S2: Data of the family 667. Chromatograms showing wild type (2A), hemizygous NM_000307.4:c.707A>C (p.(Glu236Ala)) mutation in the affected male (2B) and c.707A>C (p.(Glu236Ala)) mutation in the female carrier (2C). One affected male was in the family (2D).

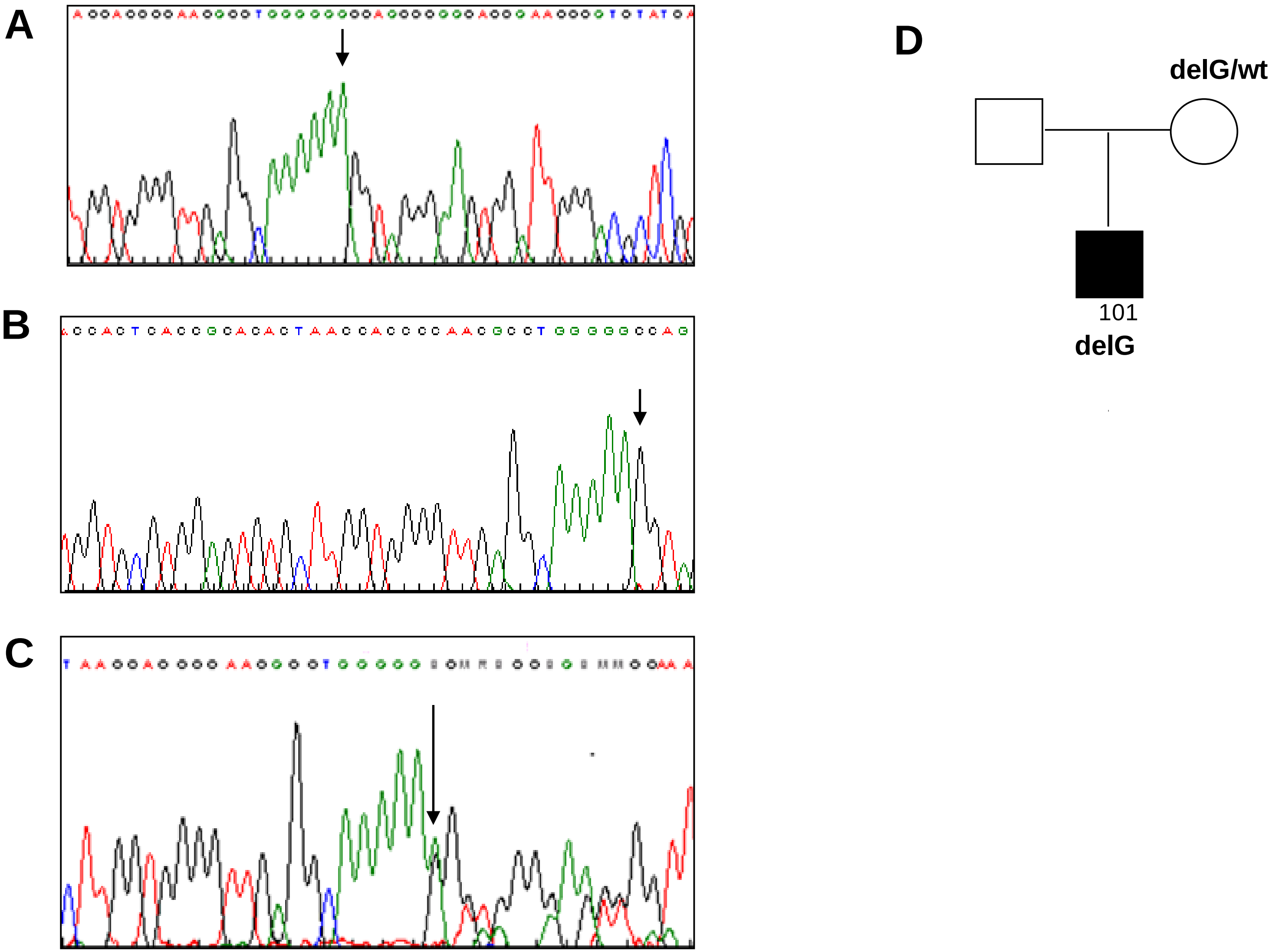

<u>Figure S3</u>: Data of the family 572. Chromatograms showing wild type (3A), hemizygous NM_000307.4:c.346delG (p.(Ala116Profsx26)) mutation in the affected male (3B) and c.del346G (p.(Ala116Profsx26)) mutation in the female carrier (3C). One affected male was in the family (3D).

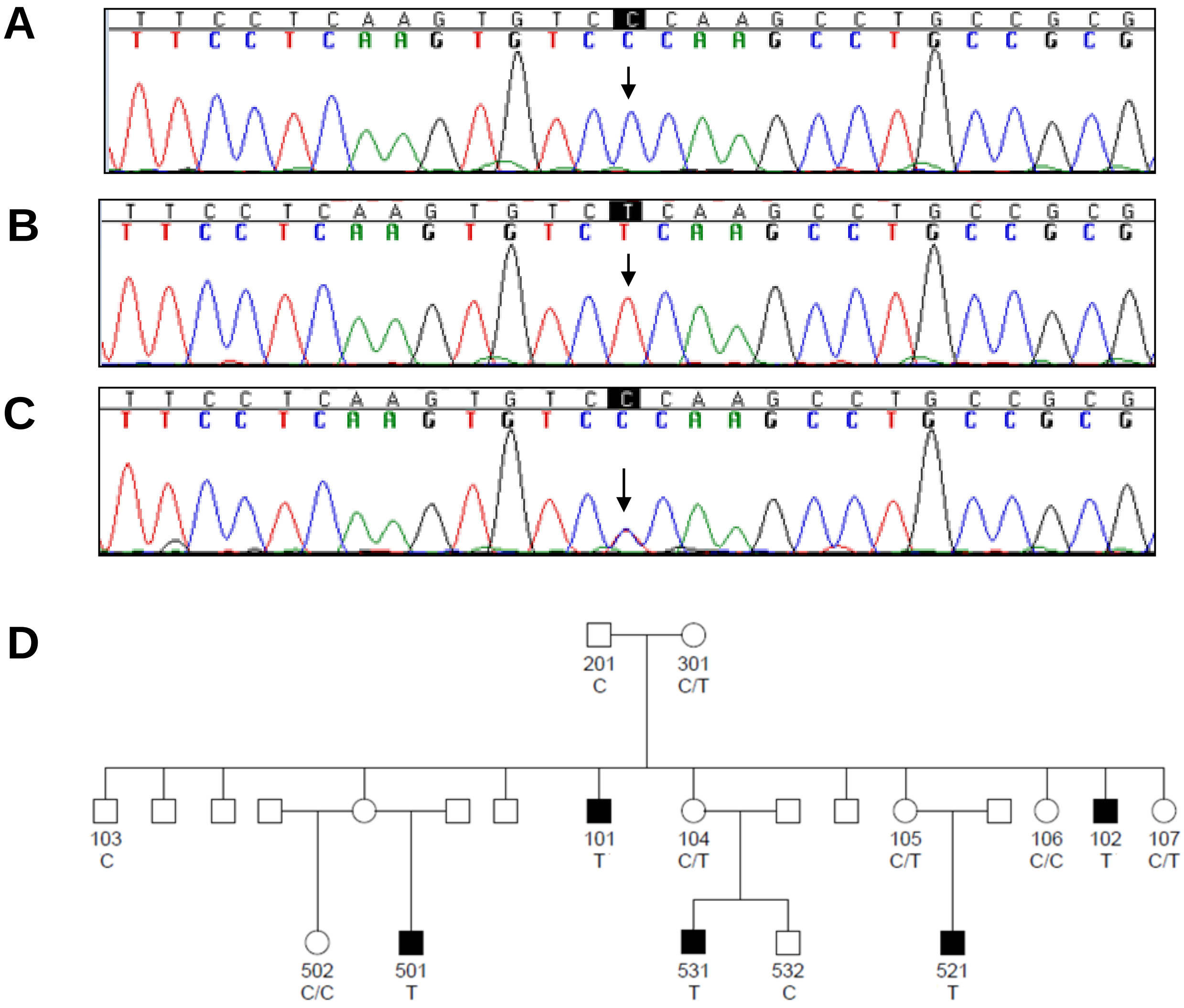

<u>Figure S4</u>: Data of the family 1225. Chromatograms showing wild type (4A), hemizygous NM_000307.4:c.902C>T (p.(Pro301Leu)) mutation in the affected male (4B) and c.902C>T (p.(Pro301Leu)) mutation in the female carrier (4C). The pedigree contains five affected males and is consistent with X-linked recessive inheritance (4D).

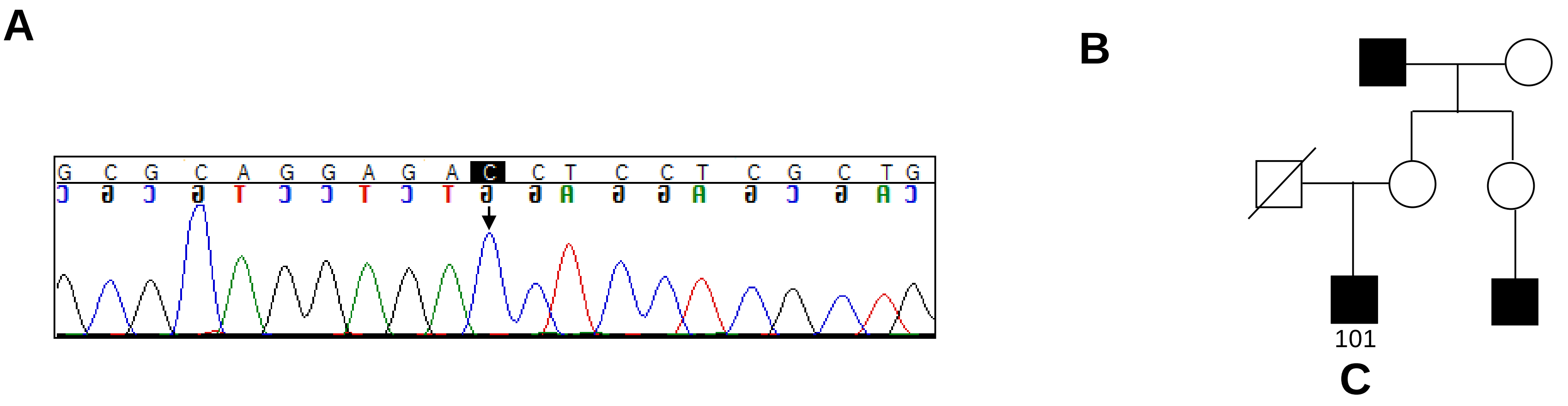

<u>Figure S5</u>: Data of the family 1535. Chromatogram showing hemizygous NM_000307.4:c.987T>C (p.(Ile308Thr)) mutation in the affected male (5A) There are three affected males and the pedigree is consistent with X-Linked recessive inheritance (5B).
